# Characterization of the *cpt1b* Gene in Response to a Tributyrin-Supplemented Diet: Cloning, Tissue-Specific Expression, and Intestinal Metabolic Function in Mandarin Fish (*Siniperca chuatsi*)

**DOI:** 10.3390/cimb48030305

**Published:** 2026-03-12

**Authors:** Er-Xue Xu, Yi Guo, Yi-Huan Xu, Teng-Fei Bao, Cheng-Bin Wu, Xiao-Wei Gao, Chun-Guang Gong

**Affiliations:** 1 Department of Biology and Health, Chizhou Vocational and Technical College, Chizhou 247000, China; czyxex@sohu.com (E.-X.X.);; 2 Ocean College, Hebei Agricultural University, Qinhuangdao 066003, China; gy020302@163.com (Y.G.);; 3 Hebei Key Laboratory of Aquaculture Nutritional Regulation and Disease Control, Qinhuangdao 066003, China

**Keywords:** *Siniperca chuatsi*, *cpt1b*, tributyrin, molecular cloning

## Abstract

Tributyrin (TB), as a novel feed additive, holds broad market prospects and is crucial for promoting fish growth and maintaining intestinal health. We first identified the fatty acid metabolism-related gene *cpt1b* in the intestines of mandarin fish (*Siniperca chuatsi*) from the TB-supplemented group. A total of 600 mandarin fish (200.0 ± 5.0 g) were evenly allocated into three groups. The control group (C) received only the standard extruded feed, while the experimental groups were supplemented with tributyrin (TB) at concentrations of 500 mg/kg (T1 group) and 1000 mg/kg (T2 group), respectively. Cloning yielded a 2364 bp open reading frame (ORF) encoding 787 amino acids, with the gene possessing two conserved transmembrane domains. Phylogenetic analysis further indicated a close phylogenetic relationship between largemouth blackbass (*Micropterus salmoides*) and mandarin fish. Tissue distribution and intestinal enzyme activity analyses revealed that supplementation with varying concentrations of TB upregulates *cpt1b* gene expression in different tissues, while modulating intestinal digestive enzyme and antioxidant enzyme activities. Our findings suggest a potential mechanism involving enhanced intestinal enzyme activity, reduced fat accumulation, increased expression of lipid oxidation-related genes, and accelerated TB degradation in the intestine.

## 1. Introduction

Fish growth and health are closely related to intestinal enzyme activity and gut morphology [1], and the integrity of gut structure is essential for digestive and absorptive function [2]. Carnivorous fish generally exhibit higher protease and lipase activities than other trophic types, which are strongly influenced by diet composition and gastrointestinal structure. Mandarin fish (*Siniperca chuatsi*), a typical carnivorous species, naturally preys on live baitfish but can be gradually domesticated to accept formulated artificial feed [3]. Domesticating mandarin fish through artificial feeding expands their aquaculture scale and reduces the ecological damage caused by harvesting large quantities of baitfish [4]. However, formulated feeds contain ingredients such as starch and plant proteins that are difficult for carnivorous fish to fully digest, and they differ substantially from live baitfish in nutritional composition. These differences may impair growth performance and even affect overall fish health. Studies have shown that under formulated feeding regimes, both hybrid mandarin fish (*Siniperca chuatsi* × *Siniperca scherzeri*) and mandarin fish exhibit slowed growth rates [5], reduced thickness of the gastric submucosal and muscular layers, decreased branching of intestinal villi, and diminished intestinal protease activity [4,6]. To improve this situation, butyric acid has attracted considerable attention as a vital energy source for intestinal epithelial cells, promoting intestinal development and thereby enhancing feed digestion and absorption [7,8]. Extensive in vitro and in vivo studies indicate that butyrate can improve lipid and glucose metabolism, enhance energy homeostasis, and modulate immune function in mammals [9,10]. In fish, multiple studies have indicated that butyric acid can enhance growth performance, improve intestinal health, and boost non-specific immunity [11,12,13]. However, butyric acid is highly volatile and possesses an irritating odor, making it difficult to incorporate into aquatic feed. Furthermore, it is readily digested and absorbed before reaching the small intestine, hindering its delivery to the hindgut where it could exert its physiological effects. These drawbacks significantly limit the adoption and application of butyric acid in aquaculture feed. To address this industry challenge, butyrate salts have been added to feed as an alternative. However, these salts are prone to deliquescence, which impedes their widespread use [14]. Tributyrin (TB), a butyric acid precursor composed of *N*-butyric acid and glycerol, has emerged as an effective solution. Upon entering the intestine, this short-chain fatty acid is hydrolyzed by pancreatic lipase into butyric acid, which is rapidly absorbed by intestinal epithelial cells. It possesses excellent palatability, delivers the same physiological effects as butyric acid, and readily reaches target sites to exert its functions, effectively bypassing the stomach to reach the intestines and enter systemic circulation [15], where it acts on organs such as the liver, muscles, and adipose tissue. TB addresses the challenges of incorporating butyric acid into feed, including its liquid state at room temperature, high volatility, and pungent odor, while also mitigating the hygroscopic nature of butyrate salts. As such, TB presents broad research prospects.

Fatty acid metabolism, particularly the beta-oxidation pathway, plays a critical role in tissue proliferation, energy production, and cell growth [16]. Following the administration of TB-supplemented feed to mandarin fish, our intestinal transcriptome analysis identified a set of differentially expressed genes (DEGs) involved in fatty acid metabolism pathways [17]. Among these DEGs, carnitine palmitoyltransferase 1B (CPT1B) was prioritized as a key target due to its well-documented role as a rate-limiting enzyme in mitochondrial fatty acid β-oxidation [18], which is closely related to our hypothesized metabolic regulatory effects of TB [17]. Fatty acid oxidation is a central catabolic pathway utilized by diverse eukaryotic cells to provide energy for mitochondria. Cellular long-chain fatty acid oxidation in mitochondria is facilitated by the regulated translocation of activated fatty acids from the cytoplasm to the mitochondrial matrix through the coordinated action of two enzymes: CPT1, located on the outer mitochondrial membrane facing the cytoplasm, and CPT2, located on the inner mitochondrial membrane facing the matrix. CPT1 catalyzes the conversion of long-chain acyl-coenzyme A (CoA) to long-chain acylcarnitines, which then traverse the inner mitochondrial membrane via carnitine/acylcarnitine translocase. Subsequently, CPT2 regenerates long-chain acyl-CoA from acylcarnitine, enabling fatty acid oxidation to yield acetyl-CoA. The regulated step in fatty acid oxidation occurs at *cpt1* [19], which can regulate fatty acid oxidation according to the energy demands of the organism, effectively maintaining a balanced state of fat metabolism, and is therefore also known as an energy sensor. Among the various CPT1 isoforms, the most extensively characterized in mammals and teleosts are the hepatic isoform CPT1A (CPT1α) and the muscular isoform CPT1B (CPT1β), both of which were initially identified in rats [20,21]. Gutieres et al. [22] successfully cloned the full-length cDNA sequence of the *cpt1* gene in rainbow trout (*Oncorhynchus mykiss*), providing the first evidence of *cpt1* gene presence in fish tissues, though without determining the precise number of its subtypes. Subsequent investigations in rainbow trout [23] and yellow catfish (*Pelteobagrus fulvidraco*) [24] revealed five distinct *cpt1* isoforms—*cpt1α1a*, *cpt1α1b*, *cpt1β1a*, *cpt1β1b*, and *cpt1α2*—and four isoforms—*cpt1α1a*, *cpt1α1b*, *cpt1α2a*, and *cpt1β*, respectively. These findings indicate that a genome duplication event may have driven the diversification of the *cpt1* gene family in fish [23]. Subsequent extensive studies on *cpt* genes in numerous fish species revealed high homology with mammalian *cpt* genes, with relatively conserved amino acid sequences across mammals and teleosts. However, research on the functional characterization and regulatory mechanisms of the CPT family within mandarin fish remains scarce in both domestic and international fields.

To this end, the present study first cloned the coding region of the *cpt1b* gene in mandarin fish, obtained its open reading frame (ORF) sequence, and performed bioinformatics analysis. Concurrently, by integrating existing vertebrate genomic data, we investigated the sequence conservation of the *cpt1b* gene across vertebrates to elucidate the evolutionary relationship between CPT proteins in fish and other vertebrates. Building on this foundation, we further analyzed the effects of dietary TB supplementation on intestinal digestive enzyme activities and *cpt1b* gene expression levels in different experimental groups of mandarin fish. Notably, as a precursor of butyrate, TB may exert its regulatory effects through butyrate-mediated activation of signaling pathways such as AMPK, leading to upregulated *cpt1b* gene expression in tissues including the intestine and liver. This, in turn, could enhance β-oxidation of long-chain fatty acids, ultimately improving intestinal digestive function and systemic lipid metabolic homeostasis. This proposed regulatory mechanism requires experimental validation. The findings of this study will provide theoretical support for elucidating the molecular mechanism by which TB regulates intestinal function and lipid metabolism in mandarin fish through modulation of *cpt1b* gene expression.

## 2. Materials and Methods

### 2.1. Animal Ethics

This study was conducted following the Laboratory Animal Welfare Guidelines of China (GB/T 35892-2018), and the experiment was approved by the Animal Experimentation Ethics Committee of Hebei Agricultural University (Grant No. 2023036).

### 2.2. Fish Sampling

The experimental mandarin fish (*Siniperca chuatsi*) were supplied by Anhui Xinong Ecological Aquaculture Co., Ltd. (Hekou Village, Qidu Town, Shitai County, Chizhou City, Anhui Province, China). Prior to the commencement of the study, dietary conversion of mandarin fish had been successfully accomplished. A total of three hundred healthy, disease-free mandarin fish with robust feeding capacity, each weighing approximately 200 g (average body weight of 200.0 ± 5.2 g), were selected for the experiment. The fish were evenly allocated into three groups and reared in net cages (1.5 m × 1.5 m × 1.2 m), one hundred fish per group (*n* = 100), with mandarin fish extruded feed provided throughout an eight-week trial period. The control group (C) received only the standard extruded feed, while the experimental groups were supplemented with tributyrin (TB) at concentrations of 500 mg/kg (T1 group) and 1000 mg/kg (T2 group), respectively, then administered twice daily (7:00 a.m. and 5:00 p.m.). Throughout the rearing period, water quality parameters were maintained as follows: temperature at 25 ± 1 °C, pH 7.8–8.2, and dissolved oxygen ≥ 5.0 mg/L. After two months of feeding, five fish from each group were randomly selected and dissected following anesthesia with MS-222 (100 mg/L; Cat. D0063637, Shanghai Amperexperiment Technology Co., Anpel, Shanghai, China). The brain, intestine, liver, and muscle tissues were excised on ice, immediately frozen in liquid nitrogen, and subsequently stored at −80 °C for further analyses.

### 2.3. Total RNA Isolation and cDNA Synthesis

The total RNA was isolated using TRIzol reagent (Cat. 15596026, Invitrogen, Carlsbad, CA, USA) following the method described by Zhang et al. [25]. Total RNA was extracted from each sample. The quality and concentration of total RNA were assessed using 1% agarose gel electrophoresis and a micro-volume nucleic acid and protein detector (Monad GD30102, Suzhou, China). Reverse transcription was performed using the HiScript^®^ III 1st Strand cDNA Synthesis kit (Catalog No.: R312-01, VAZYME, Nanjing, China) to synthesize cDNA template strands.

### 2.4. Cloning of the cpt1b Gene in Mandarin Fish

**Figure 1:**
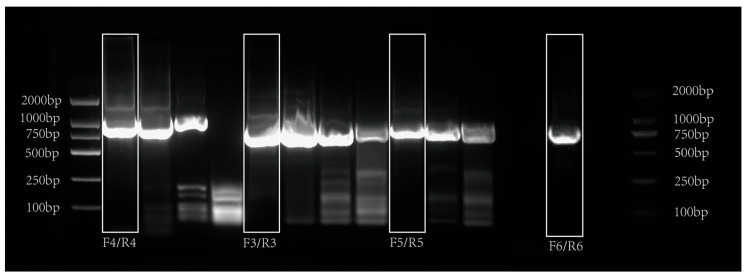
PCR-specific amplification results in mandarin fish (*Siniperca chuatsi*) intestines.

Based on partial gene sequences retrieved from the mandarin fish genome database, combined with transcriptome sequencing data generated in our laboratory and the reference sequence of the mandarin fish cpt1b gene (NCBI Accession No.: XM_044192898.1). The amplifications were performed using 2X FidCycle Fast High Fidelity PCR Mix (containing blue dye) (Product No.: B639300, BBI, Shanghai, China), and multiple pairs of nested PCR primers were designed using Primer5 software for the cloning of the cpt1b gene coding region. All primers were synthesized by the Sequencing Department of Sangyo Biotech Co., Ltd. (Beijing, China), with detailed information listed in Table S1. For cloning of the cpt1b coding region, due to its large fragment size, we adopted a strategy of “segmented amplification followed by overlapping extension PCR (OE-PCR) assembly” to obtain the full-length coding sequence (CDS). Specifically, the complete CDS of cpt1b was divided into two overlapping segments (Segment 1 and Segment 2). The first round of nested PCR was performed using cDNA from mandarin fish intestine as templates, with two pairs of specific outer primers: F1/R1 for Segment 1 and F2/R2 for Segment 2. After amplification, 1.0% agarose gel electrophoresis showed multiple diffuse bands or no distinct target bands. To improve the amplification specificity and sensitivity, the first-round PCR products were diluted 100-fold as templates for the second round of nested PCR. Specifically, the inner primer pairs F3/R3 and F4/R4 were designed to target the specific region of Segment 1 (amplified by F1/R1 in the first round), and the inner primer pairs F5/R5 and F6/R6 were designed for the specific region of Segment 2 (amplified by F2/R2 in the first round). After the second-round amplification, 1.0% agarose gel electrophoresis was performed again, and four distinct bands with sizes consistent with the expected target fragments were obtained (as shown in Figure 1). Subsequently, the four target bands were separately purified using the EZNA Gel Purification Kit (Omega, Norcross, GA, USA). The purified fragments were sent for sequencing, and the obtained sequences were assembled using DNAMAN software (version: 9.0.1.116). BLAST alignment against the NCBI reference sequence (XM_044192898.1) showed that the assembled full-length CDS had a similarity of 99.96%.

The thermal cycling programs for first-round nested PCR and second-round nested PCR are detailed in Table S1.

### 2.5. Data Processing and Analysis of Results

The physicochemical properties and structural characteristics of CPT1B in mandarin fish were predicted using online analytical tools (Table S2). Amino acid sequences of CPT1 from other fish species were retrieved from the NCBI database, and protein similarity percentages were calculated with MEGA 11.0 software in conjunction with the BLAST function of NCBI. Multiple sequence alignments of CPT1 proteins were performed using the ClustalW algorithm within the DNAMAN package. A phylogenetic tree was then constructed in MEGA 11.0 employing the neighbor-joining (NJ) method under the JTT+G model [26], with confidence levels assessed through 1000 bootstrap replicates. Furthermore, comparative genomic analyses were conducted to examine the structural organization of *cpt1b* genes across different vertebrates. Protein sequences were subsequently submitted to the SWISS-MODEL server for three-dimensional structural prediction, and the resulting PDB files were processed with PyMOL (version: 3.1.6.1) software to generate 3D protein structure models.

### 2.6. Tissue Distribution Levels of cpt1b in Mandarin Fish

The mRNA expression level of *cpt1b* in different tissues was quantified using quantitative real-time PCR (qPCR). cDNA from each tissue was used as the template, and detection was performed using the SYBR Green I method, with *β-actin* as the internal reference. Specific primers were designed using the online website Primer3Plus. Gene-specific primers were synthesized by Sangon Biotechnology Co. (Beijing, China) (Table S1). There were three biological replicates per group (*n* = 3). Each 10 μL reaction system was prepared in a 96-well plate following the protocol of the ChamQ Universal SYBR qPCR Master Mix (Novozymes, Nanjing, China). The amplification program was set as follows: 98 °C for 3 min, followed by 39 cycles of 95 °C for 10 s and 60 °C for 30 s for annealing and extension. Relative expression levels across tissues were calculated using the 2^−ΔΔCT^ method.

### 2.7. Intestinal Enzyme Activity

Homogenize the intestines in frozen physiological saline, centrifuge, and collect the supernatant for analysis of lipase (LPS), amylase (AMS), trypsin (TRY), superoxide dismutase (SOD), and catalase (CAT) activities. Three replicates were performed for each indicator. Commercially available kits (Nanjing Jiancheng Bioengineering Institute, Nanjing, China) were used for analysis, with the testing procedures strictly adhering to the manufacturer’s instructions.

### 2.8. Statistical Analysis

Enzyme activity data were analyzed using one-way ANOVA in SPSS (version: R26.0.0.0), with Tukey’s HSD Test and Duncan’s Test applied to assess significant differences. qPCR data underwent two-way ANOVA followed by Duncan’s multiple range test. Results are presented as mean ± standard deviation (mean ± SD). Differences were considered significant at *p* < 0.05. Graphs were generated using GraphPad Prism 10 (GraphPad Software, San Diego, CA, USA) for graphical representation.

## 3. Results

### 3.1. Molecular Cloning and Characterization of CPT1B

**Figure 2:**
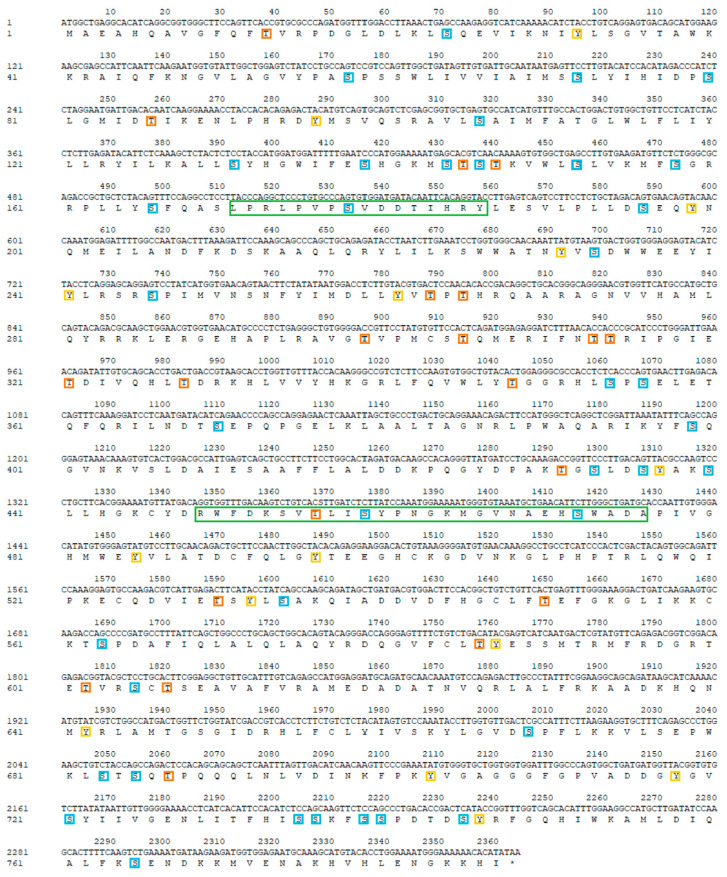
Nucleotide sequence encoding CPT1B in mandarin fish (*Siniperca chuatsi*) and the deduced amino acid sequence. Positions of the nucleotide and amino acid (left number), two lipoylcarnitine-binding sites (green solid box), putative serine phosphorylation sites (blue solid boxes), tyrosine phosphorylation sites (orange solid boxes), and threonine phosphorylation sites (yellow solid boxes). *: Terminator codon.

Analysis of the amino acid sequence of mandarin fish CPT1B revealed that the open reading frame (ORF) spans 2364 bp. Its physicochemical properties, predicted using the ExPASy ProtParam tool, indicate that mandarin fish CPT1B is a relatively stable protein with partial hydrophilicity, expected to encode 787 amino acids. The predicted molecular weight of CPT1B is 89.44 kDa, the instability index is 39.30 (II < 40), and its predicted theoretical isoelectric point is 8.81 (PI > 7), indicating that it is a stable alkaline protein. TMHMM--transmembrane prediction server analysis revealed two transmembrane domains in the amino acid sequence of the mandarin fish CPT1B protein, located in the regions 64–84 and 104–126, with no signal peptides present, with no signal peptide present. Prediction of phosphorylation sites using NetPhos 3.0 revealed thirty-eight serine phosphorylation sites, twenty-one threonine phosphorylation sites, and sixteen tyrosine phosphorylation sites (Figure 2).

**Figure 3:**
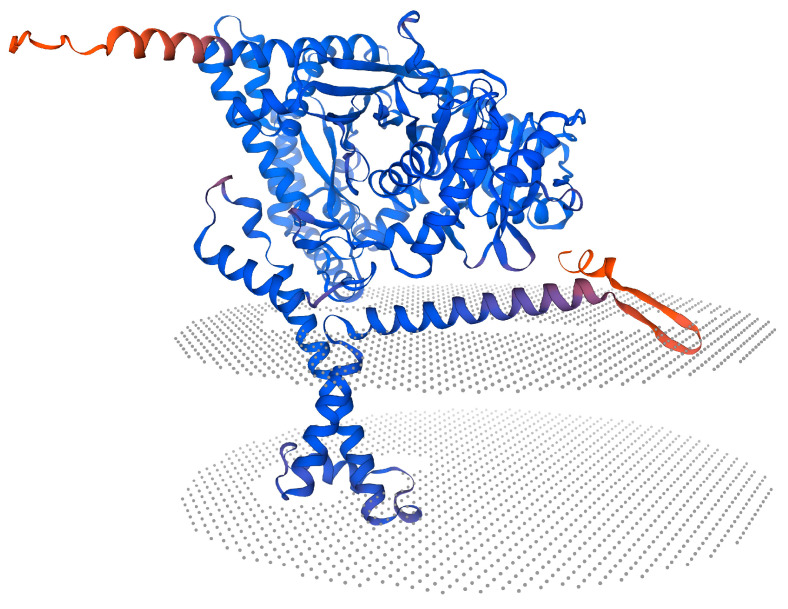
Tertiary structure prediction of CPT1B protein in mandarin fish. The cartoon shows CPT1B embedded in the mitochondrial outer membrane (gray dotted planes): N- and C-terminal transmembrane helices (orange/red) form the membrane anchor, while the catalytic core (blue) extends into the cytosol.

NetNGlyc-1.0 prediction of *N*-glycosylation sites identified two potential sites in the mandarin fishCPT1B amino acid sequence, located at positions 312 (NTTR) and 367 (NDTS). CDD analysis revealed the presence of a single *N*-terminal structural domain in mandarin fishCPT1B, while PROSITE prediction indicated two putative lipoylcarnitine-binding regions within the amino acid sequence, spanning residues 171–186 and 449–476. Subcellular localization analysis performed with Euk-MpLoc 2.0 indicated that mandarin fish CPT1B is predominantly associated with microsomes and mitochondria. Prediction of the protein’s secondary structure using SOPMA showed that mandarin fish CPT1B consists of α-helices (46.00%), extended strands (10.93%), and irregular coiled coils (43.07%). The tertiary structure of mandarin fish CPT1B, modeled by SWISS-MODEL (Figure 3).

### 3.2. CPT Phylogenetic Analysis

**Figure 4:**
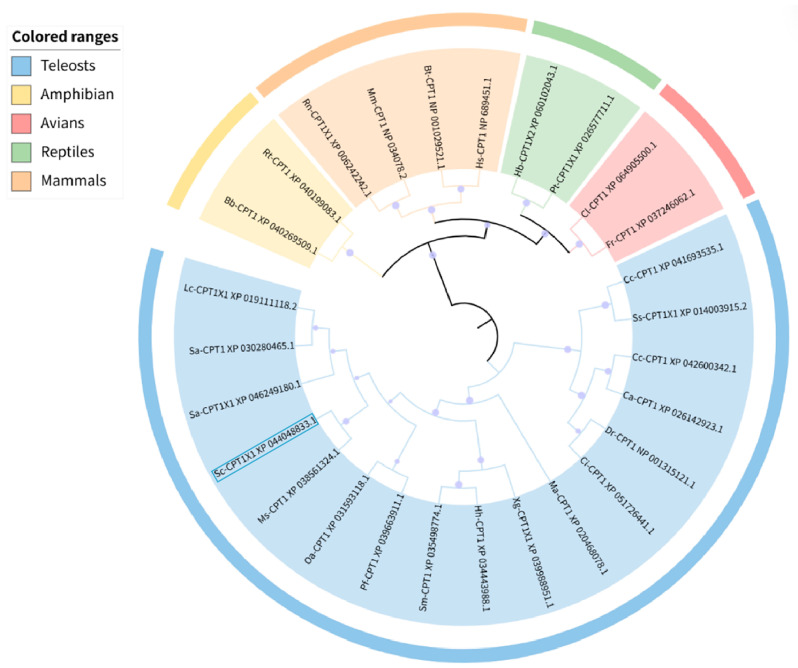
Phylogenetic tree based on the amino acid sequence of CPT1B in mandarin fish and in other vertebrate species. The mandarin fish is highlighted with a blue box (the species and their serial numbers are listed in Supplementary Table S3). Purple dots: represents the self-exposure value, with different magnitudes indicating the magnitude of the self-exposure value.

The amino acid sequence of CPT1B was subjected to homology analysis using BLAST in NCBI to identify species with the highest sequence similarity to mandarin fishCPT1B. A phylogenetic tree was subsequently constructed with MEGA 11.0 employing the neighbor-joining (NJ) method. The results revealed that the tree was divided into two principal clades: one comprising teleosts, in which CPT1 from Atlantic salmon (*Salmo salar*), lake whitefish (*Coregonus clupeaformis*), grass carp, zebrafish, crucian carp (*Carassius auratus*), and common carp clustered together, forming a lineage that was evolutionarily distant from fishes of other families. The second clade included amphibians, birds, mammals, and reptiles. Moreover, it is evident that the mandarin fish CPT1B shares a close phylogenetic relationship with the largemouth blackbass *(Micropterus salmoides)* (Figure 4).

### 3.3. Construction and Structural Analysis of the Linkage Map of the *cpt1b* Gene in Vertebrates

**Figure 5:**
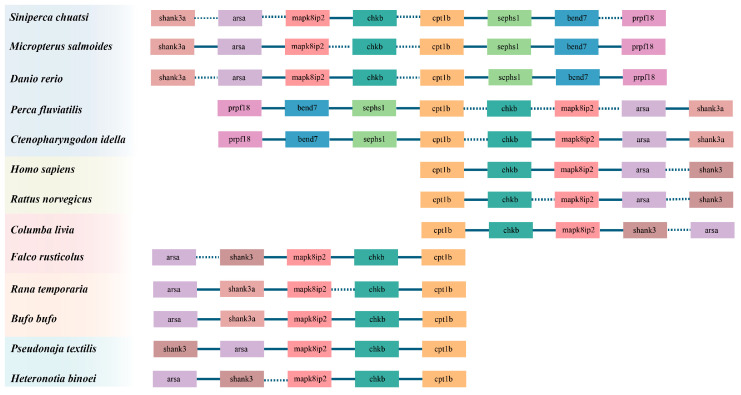
*cpt1* gene synteny comparisons in different vertebrate genomes. The colorful blocks indicate intergenic regions; the solid and dotted lines indicate regions without genes. The different gradient blocks on the left represent different classes.

Through collinearity analysis of teleosts (mandarin fish, largemouth blackbass, zebrafish (*Danio rerio*), European perch (*Perca fluviatilis*), grass carp *(Ctenopharyngodon idella*), amphibians (common frog (*Rana temporaria*), common toad (*Bufo bufo*), avians (rock pigeon (*Columba livia*), gyrfalcon (*Falco rusticolus*), mammals (human (*Homo sapiens*), Norway rat (*Rattus norvegicus*)), and reptiles (Eastern Brownsnake (*Pseudonaja textilis*), rickly gecko (*Heteronotia binoei*), we observed that *cpt1b*, *shank3/3a*, *arsa*, *mapk8ip2*, and *chkb* consistently appeared across all vertebrate species examined. Despite variations in gene order among different clades, the “collinearity association” of these five genes remained intact, demonstrating high structural conservation. Furthermore, we found that *sephs1*, *bend7*, and *prpfl8* were consistently present in teleosts but absent in other vertebrate species, indicating group-specific evolutionary divergence (Figure 5).

**Figure 6:**
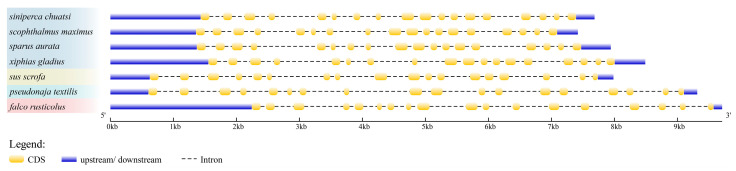
The exon–intron structure of *cpt1b*. The yellow box and violet lines represented CDS and upstream/downstream, respectively. CDS: coding sequences; kb: kilobase pairs. The different gradient blocks on the left represent different classes.

Analysis of the *cpt1b* gene structure in vertebrates revealed that the number of coding sequences (CDS) remained consistent across the examined species, with only minor variations in length. The coding regions of this gene exhibited high conservation across species, whereas non-coding regions, such as introns, showed significant species-specific differences in length. This resulted in substantial variations in total sequence length among different species, highlighting the evolutionary plasticity of non-coding regions (Figure 6).

### 3.4. Comparison of CPT Protein Sequences

**Figure 7:**
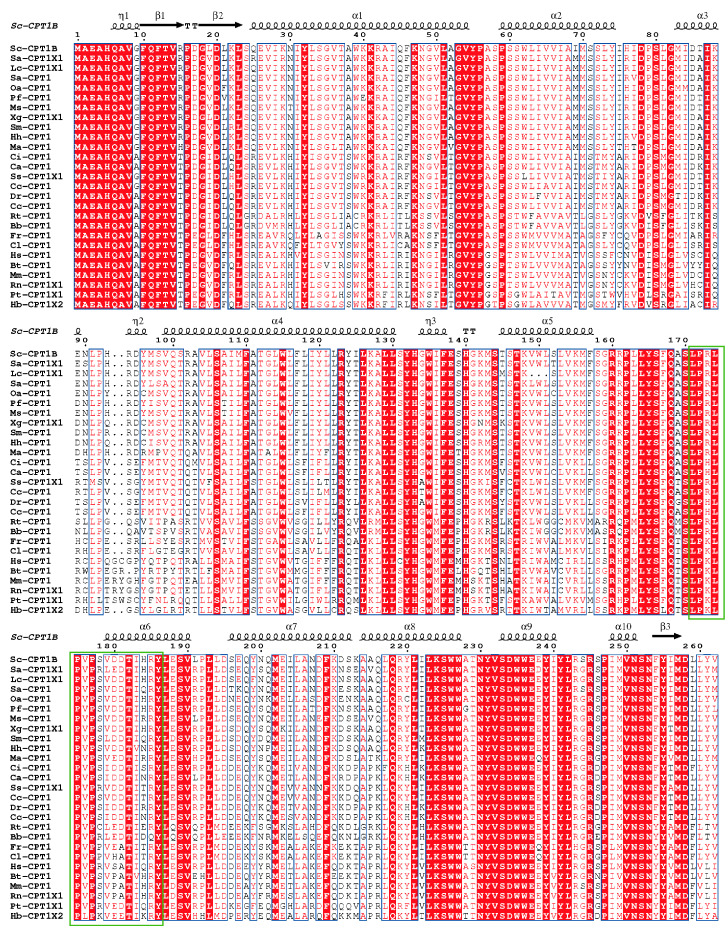

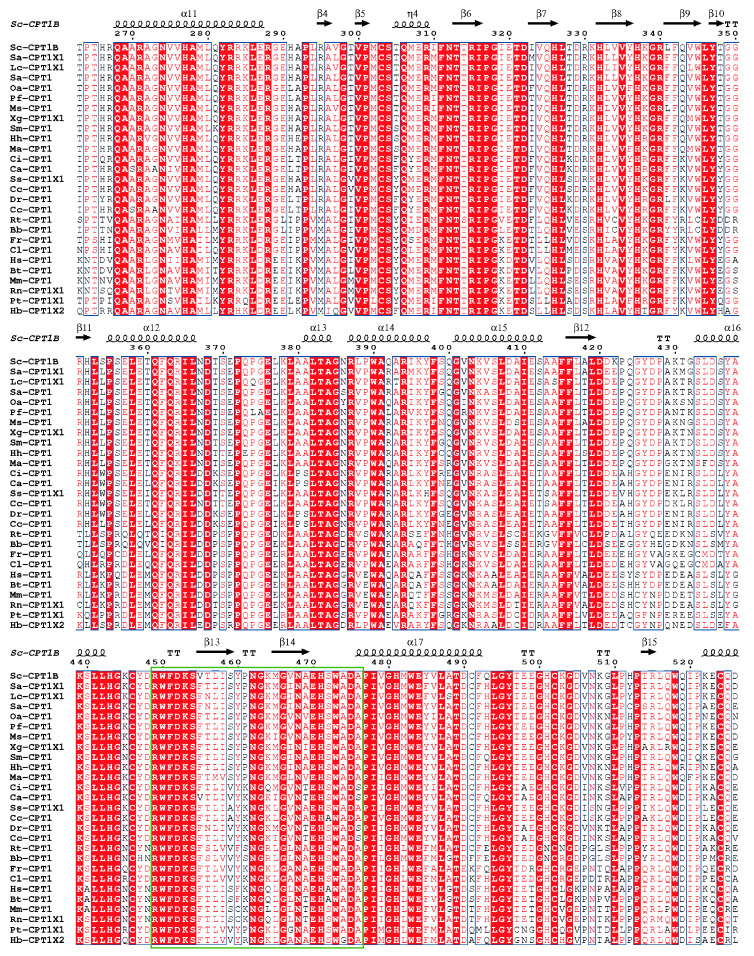

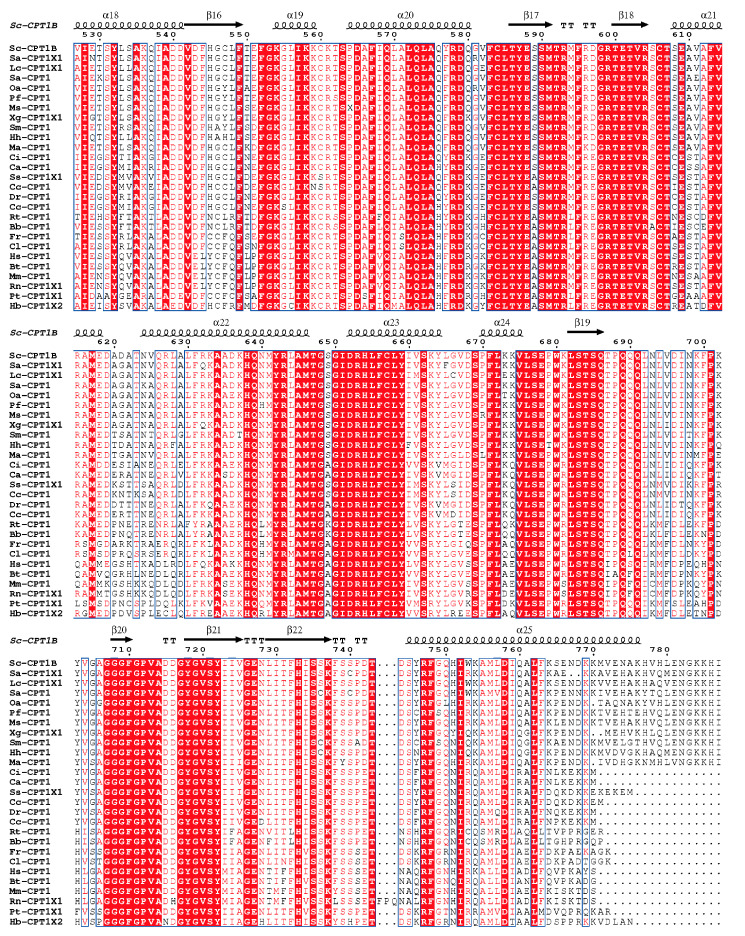
Alignment of CPT1B amino acid sequence of mandarin fish with that of different species. Solid green boxes indicate conserved lipoylcholine binding site (the species and their serial numbers are listed in Supplementary Table S4). White text on a red background: Strict identity; Red text on a white background: High similarity; Blue frame: Block of similarity; Black text on a white blackground: No similarity.

The results of amino acid sequence comparison between mandarin fish CPT1B and CPT proteins from other species are presented in Figure 7. The analysis revealed that CPT1B is highly conserved among vertebrates, exhibiting two homologous lipoylcarnitine-binding domains. Homology assessment demonstrated that mandarin fish CPT1B shares strong similarity with largemouth blackbass (95.30%). Moreover, CPT1 exhibits over 60% homology with mammals, amphibians, reptiles, birds, and other teleosts, underscoring the high degree of evolutionary conservation of this protein across diverse taxa.

### 3.5. Tissue-Specific Distribution of cpt1b Gene Expression in Mandarin Fish

**Figure 8:**
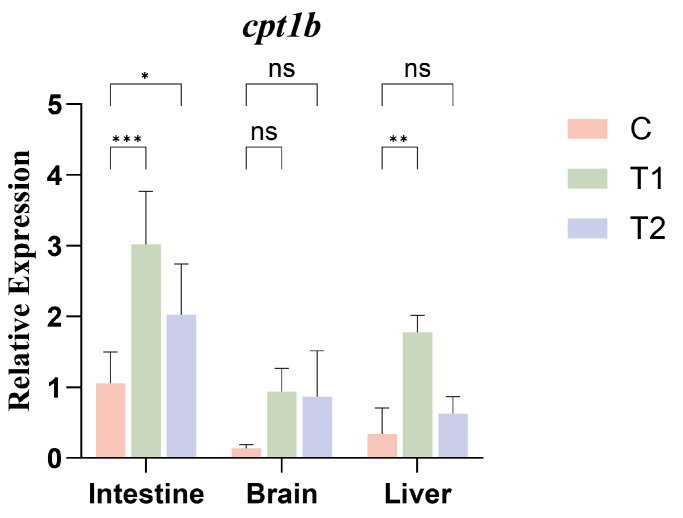
Tissue distribution levels of the *cpt1b* gene. Error bars represent the standard deviation (SD); *n* = 3 biological replicates (*n* = 3). *: significant difference (*p* < 0.05); **: the difference is very significant. (*p* < 0.01); ***: the difference is extremely significant. (*p* < 0.001); ns: no significant difference (*p* > 0.05).

To investigate the expression of the *cpt1b* gene in various tissues of mandarin fish, qPCR was employed to detect expression levels in the intestine, liver, and brain tissues, with results shown in Figure 8. The *cpt1b* gene was expressed in all tissues but at varying levels. The addition of TB to feed induced a response in *cpt1b* gene expression in the intestine and liver, while no significant difference was observed in brain tissue. Concurrently, the results indicate that adding different concentrations of triacylglycerol significantly influenced the expression levels of the *cpt1b* gene in various tissues of the mandarin fish. Among these, only the T1 concentration showed a significant enhancing effect on liver expression. The intestine exhibited greater sensitivity to the fatty acid oxidation metabolism of TB at the T1 concentration, with a more pronounced enhancing effect compared to T2.

### 3.6. Analysis of Intestinal Enzyme Activity in Mandarin Fish

**Figure 9:**
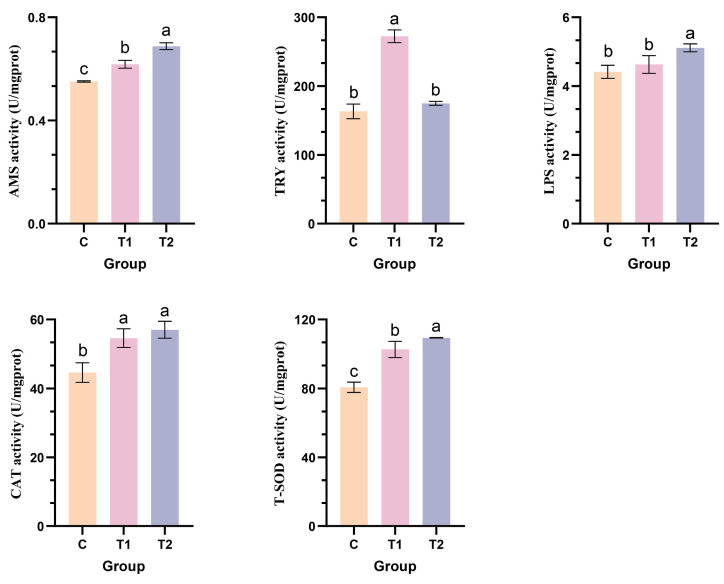
Effects of different concentrations of TB in feed on intestinal AMS (A), TRY (B), LPS (C), CAT (D), and T-SOD (E) levels in mandarin fish. Columns labeled with the same lowercase letter indicate no significant difference (*p* > 0.05), while different lowercase letters indicate significant differences (*p* < 0.05).

The effects of TB on the intestinal health of mandarin fish were further investigated by measuring the activities of digestive enzymes (AMS, TRY, LPS) and antioxidant enzymes (CAT, T-SOD). Results indicated that TB positively regulated digestive enzyme and antioxidant enzyme activities in mandarin fish, with effects varying by additional concentration. The T2 concentration significantly enhanced AMS, LPS, CAT, and T-SOD activities, while the T1 concentration most notably increased TRY activity. Adding different concentrations (T1, T2) of TB significantly influenced both AMS and T-SOD in mandarin fish, with the differences in enzyme activity becoming more pronounced as the added concentration increased. Adding TB at T1 concentration significantly affected TRY activity, while T2 concentration showed no significant difference. Conversely, for LPS activity, TB at T2 concentration had a significant effect, whereas T1 concentration showed no significant difference. Both T1 and T2 concentrations affected CAT activity, but no significant difference was observed between them (Figure 9).

## 4. Discussion

CPT is a pivotal enzyme in animal fatty acid metabolism, comprising two isoforms—CPT1 and CPT2—situated on the outer and inner mitochondrial membranes, respectively. Both isoforms are integral to the process of fatty acid β-oxidation [27,28]. Two genes encode for *cpt1*, *cpt1a* and *cpt1b*, enriched in the liver and the muscle, respectively [19], among which CPT1B is the most abundant in the heart and skeletal muscle, the protein encoded by this gene is a member of the carnitine/choline acyltransferase family, acting as a rate-limiting enzyme in the β-oxidation pathway of long-chain fatty acids within muscle mitochondria, which plays a crucial role in transferring long-chain fatty acid from CoA to carnitine for oxidation in the mitochondrion. This study first isolated the ORF sequence encoding *cpt1b* from the intestine of the mandarin fish, spanning 2364 bp, confirming the presence of the mitochondrial carnitine palmitoyl transferase system. Multiple sequence alignment and homology analysis of the CPT1B amino acid sequence from mandarin fish with other teleosts (e.g., zebrafish, Atlantic salmon) revealed significant amino acid sequence homology, indicating this gene is highly conserved throughout the evolutionary history of teleost fishes. Its core biological functions (such as regulation of fatty acid β-oxidation transport) may exhibit consistency across species.

Our research has found that *cpt1b* exhibits a conserved gene structure characterized by the maintenance of exon and intron numbers, while intron lengths show significant variation. This indicates that *cpt1b* gene may have undergone distinct evolutionary trajectories across different species. Furthermore, *cpt1b* genes across different taxonomic groups exhibit chromosomal collinearity (i.e., conserved patterns of adjacent gene arrangement upstream and downstream), corroborating the stability of its exon–intron framework. It has been confirmed that even when gene order is not perfectly aligned, *chkb*, *arsa*, and *shank3a* are consistently found in the vicinity of *cpt1b* across many studied species, providing strong support for their shared origin [18]. The findings of this study are highly consistent with previously reported evolutionary patterns of the vertebrate *cpt1b* gene, indicating that this structural pattern is universal across vertebrate lineages. This further highlights the high conservation of the core functional region of the *cpt1b* gene and the evolutionary plasticity of its non-coding regions (exons).

Protein post-translational modifications are highly diverse, with glycosylation and phosphorylation serving as key regulatory mechanisms for protein function. Glycosylation enhances the recognition specificity of proteins, not only providing targeting signals for protein sorting and transport but also optimizing biological functions by stabilizing protein conformation [29]. This study identified two *N*-glycosylation sites in the mandarin fish CPT1B protein. It is hypothesized that this modification may safeguard the physiological function of CPT1B through a dual mechanism: first, by mediating its targeted transport to mitochondria, and second, by stabilizing the protein’s own structure, ultimately ensuring the efficient role of CPT1B in fatty acid oxidation for energy supply. Furthermore, we found that the mandarin fish CPT1B protein contains abundant phosphorylation sites, including 38 serine sites, 21 threonine sites, and 16 tyrosine sites. These three types of sites achieve functional synergy through their distinct structural characteristics: phosphorylation of serine can allosterically modulate proteins and activate enzymatic activity. Threonine and serine are common phosphorylation sites, as the hydroxyl groups at their structural termini are highly reactive and can bind to phosphate groups, participating in various physiological and biochemical reactions. Tyrosine phosphorylation can also induce protein allostery and activate protein activity. Moreover, it facilitates protein binding, promoting interactions between the bound protein and other proteins to form complex multi-protein complexes, thereby further enhancing protein phosphorylation [30]. In summary, the abundant phosphorylation sites in the mandarin fish CPT1B protein suggest that it may achieve multifunctional regulation through combinatorial phosphorylation at different sites (i.e., selective phosphorylation combinations at specific sites). This enables both activation of its own enzymatic activity and mediation of specific protein–protein interactions, ultimately fine-tuning the rate of fatty acid transmembrane transport. Consequently, CPT1B can flexibly respond to dynamic changes in the body’s energy demands. In addition, we conducted phylogenetic analyses to elucidate the evolutionary relationships of *cpt1* across vertebrates. The results revealed that the *cpt1* gene segregates into two principal clades: one comprising teleosts and the other encompassing mammals, reptiles, amphibians, and birds. Within the teleost lineage, further subdivision into two closely related branches was observed, while scleractinians likewise formed two adjacent sub-branches, a pattern consistent with findings on the *cpt1b* gene in swamp eel (*Monopterus albus*) [31]. These results indicate that the *cpt1b* gene is highly conserved within teleosts and likely performs comparable functions across this group.

As a key rate-limiting enzyme in the fatty acid β-oxidation pathway, the core function of CPT is to mediate the entry of long-chain fatty acids into mitochondria for oxidative breakdown, thereby participating in the regulation of whole-body lipid homeostasis in fish. Most studies have confirmed that its upregulation can reduce body fat by accelerating lipolytic metabolism [32,33]. Existing research has shown that when exogenous fatty acid intake increases or the body’s demand for fatty acids rises, the expression of the *cpt1* gene is significantly enhanced to meet the energy supply needs of fatty acid oxidation [34]. This study found that the addition of different concentrations of TB to the feed significantly upregulates the expression of the *cpt1b* gene in the intestine and liver of mandarin fish, with the intestine being more sensitive to TB concentration—a phenomenon closely related to the intestine being the primary site for nutrient digestion and absorption, where its morphological structure and digestive enzyme activity are easily regulated by external nutritional factors. In the present study, normalization was applied to the qPCR results, whereas in our previous work, relative expression levels were reported directly, and the primers employed differed between the two studies. Despite these methodological discrepancies, both investigations consistently observed upregulation of the target gene in the treatment groups relative to the control group, with significant differences detected in both the T1 and T2 groups, this suggests that the intestine may be a key target for TB’s regulatory effects.

Digestive enzyme activity is a critical indicator reflecting intestinal development, digestive capacity, and feed utilization efficiency [35], and feed additives can influence nutrient absorption efficiency in fish by modulating the structure of digestive organs and digestive enzyme activity. Previous studies have confirmed that TB can significantly increase the activity of intestinal lipase, amylase, and trypsin in fish, while also increasing villus length and improving the thickness of the muscular layer, thereby expanding the intestinal absorptive surface area [36,37]. This aligns with the regulatory effects of TB on intestinal function in mandarin fish observed in our previous study [17]. Our findings also further confirm that TB enhances the activity of digestive enzymes and antioxidant enzymes. Notably, the activation of digestive enzymes such as intestinal lipase not only improves nutrient absorption but also directly mediates the hydrolysis of TB in the intestine to produce butyrate. As an important short-chain fatty acid derived from intestinal microbial metabolism, butyrate has been shown to activate the AMPK energy-sensing pathway by modulating the intracellular AMP/ATP ratio [38]. AMPK, a core regulator of cellular energy metabolism, exerts dual regulatory effects on lipid metabolism upon activation: on one hand, AMPK phosphorylates acetyl-CoA carboxylase (ACC) [39,40], leading to reduced ACC activity [40] and thereby inhibiting fatty acid synthesis; on the other hand, activated AMPK promotes the transcription of the *cpt1b* gene [41,42], enhancing the efficiency of long-chain fatty acid entry into mitochondria for oxidative breakdown.

Based on the above experimental findings, this study proposes a testable molecular mechanism by which TB regulates lipid metabolism in mandarin fish. Specifically, TB is hydrolyzed by intestinal digestive enzymes to release butyrate, which activates the AMPK pathway in both the intestine and liver. This activation suppresses fatty acid synthesis while upregulating *cpt1b* expression to enhance fatty acid oxidation for energy production. Concurrently, TB improves intestinal morphology and digestive enzyme activity, thereby promoting the absorption of dietary fatty acids and providing sufficient substrates for oxidation—a synergistic effect of “increased substrate supply coupled with activated catabolic pathways”. In addition, TB may also support intestinal health by enhancing antioxidant enzyme activity and attenuating intestinal inflammatory responses, thereby establishing a stable intestinal microenvironment conducive to nutrient absorption and lipid metabolism regulation. This hypothesis provides a theoretical framework for understanding the nutritional regulatory mechanism of TB, although the specific molecular details warrant further experimental validation.

## 5. Conclusions

The addition of TB to feed can regulate the expression of the lipid metabolism-related gene *cpt1b* in mandarin fish, improving the activity of intestinal digestive enzymes and antioxidant enzymes and thereby maintaining intestinal health. Cloning and bioinformatics analyses of the coding region of the *cpt1b* gene indicate that this gene is highly conserved in vertebrates and possesses a relatively stable gene structure. This study provides theoretical foundations for further investigating the specific regulatory effects of TB as a feed additive on the *cpt1b* gene and its interactions with digestive functions.

## Data Availability

The original contributions presented in the study are included in the article and Supplementary Materials; further inquiries can be directed to the corresponding authors.

## Supplementary Materials

The following supporting information can be downloaded at: https://www.mdpi.com/article/10.3390/cimb48030305/s1.
