# Supplementary material for: Characterization of the cpt1b Gene in Response to a Tributyrin-Supplemented Diet: Cloning, Tissue-Specific Expression, and Intestinal Metabolic Function in Mandarin Fish (Siniperca chuatsi)
Source: Curr Issues Mol Biol. 2026 Mar 12;48(3):305. doi: 10.3390/cimb48030305 (PMC13025966; doi:10.3390/cimb48030305)
Supplement: Supplementary file 1 [file cimb-48-00305-s001.zip › cimb-4116058-supplementary.pdf]

Table S1. PCR and qPCR Primer Pairs for the cpt1b Gene in mandarin fish (*Siniperca chuatsi)*.

Gene game	Primer sequences (5′-3′)	Application	Amplification conditions
β-actin-F	ATCGCCGCACTGGTTGTTGAC	Internal reference	95°C-30s, (95°C-10s, 60°C-30s) 39cycles
β-actin-R	CCTGTTGGCTTTGGGGTTC		
cpt1b-qF	TGCCATCATGTTTGCCACTG	qPCR	95°C-30s, (95°C-10s, 60°C-30s) 39cycles
cpt1b-qR	GCCACACTTTTGTTGACGTG		
cpt1b-F1	CACTAGAGTCACACAGCGCATA	First round of PCR	98°C-5min (98°C-30s, 55°C-1min40s, 72°C-40s) 29cycles, 72°C-5min
cpt1b-R1	TTTGTTCACATCCCCTTTACAG		
cpt1b-F2	GATACATCAGAACCCCAG	First round of PCR	98°C-5min (98°C-30s, 55°C-1min40s, 72°C-40s) 29cycles, 72°C-5min
cpt1b-R2	ATATACAGTCTATGGCATAAAC		
cpt1b-F3	CACTGCCTCGCTGACA	Second round of PCR	98°C-5min, (98°C-30s, 55°C-1min, 72°C-40s) 29 cycles, 72°C-5min
cpt1b-R3	CACCAGGATTTCAAGATTAG		
cpt1b-F4	TGGCTGTTCCTCATCTAC	Second round of PCR	98°C-5min, (98°C-30s, 55°C-1min, 72°C-40s) 29 cycles, 72°C-5min
cpt1b-R4	TGTTCAGCATTTACACCC		
cpt1b-F5	TCCCTTGACAGTTACGCC	Second round of PCR	98°C-5min, (98°C-30s, 55°C-1min, 72°C-40s) 29 cycles, 72°C-5min
cpt1b-R5	AATCCACCACCAGCACCC		
cpt1b-F6	GACCGTCACCTCTTCTG	Second round of PCR	98°C-5min, (98°C-30s, 55°C-1min, 72°C-40s) 29 cycles, 72°C-5min
cpt1b-R6	ATGGCATAAACTGACAAG		

Table S2. Bioinformation analysis software.

Applications	Softwares	Websites
sequences download	NCBI	https://www.ncbi.nlm.nih.gov/
Basic local alignment search tool	BLAST	https://blast.ncbi.nlm.nih.gov/Blast.cgi
open reading frame (ORF)	ORF Finder	https://www.ncbi.nlm.nih.gov/orffinder
physicochemical properties of proteins	ExPASy	https://web.expasy.org
protein domain features	Conserved Domain Database (CDD)	https://www.ncbi.nlm.nih.gov/Structure/cdd/wrpsb.cgi
N-linked glycosylation sites	NetNGlyc-1.0	https://services.healthtech.dtu.dk/services/NetNGlyc-1.0/
Putative signal peptide predictions	SignalP-6.0 Server	https://services.healthtech.dtu.dk/services/SignalP-6.0/
Putative transmembrane regions	TMHMM	https://services.healthtech.dtu.dk/services/TMHMM-2.0/
protein secondary structures	SOPMA	https://npsa.lyon.inserm.fr/
Prediction of subcellular localization	Euk-mPLoc 2.0	http://www.csbio.sjtu.edu.cn/cgi-bin/EukmPLoc2.cgi
protein tertiary structures	Swiss-Model	https://swissmodel.expasy.org/

Table S3: Species and their sequence numbers in Figure 4.

Abbreviated names	Full names	Protein ID
Sc-CPT1B	*Siniperca chuatsi*	
Lc-CPT1X1	*Larimichthys crocea*	XP_019111118.2
Sa-CPT	*Sparus aurata*	XP_030280465.1
Sa-CPT1X1	*Scatophagus argus*	XP_046249180.1
Ms-CPT1	*Micropterus salmoides*	XP_038561324.1
Oa-CPT1	*Oreochromis aureus*	XP_031593118.1
Pf-CPT1	*Perca fluviatilis*	XP_039663911.1
Xg-CPT1X1	*Xiphias gladius*	XP_039988951.1
Sm-CPT1	*Scophthalmus maximus*	XP_035498774.1
Hh-CPT1	*Hippoglossus hippoglossus*	XP_034443988.1
Ma-CPT1	*Monopterus albus*	XP_020468078.1
Ss-CPT1X1	*Salmo salar*	XP_014003915.2
Cc-CPT1	*Coregonus clupeaformis*	XP_041693535.1
Ci-CPT1	*Ctenopharyngodon idella*	XP_051726441.1
Dr-CPT1	*Danio rerio*	NP_001315121.1
Ca-CPT1	*Carassius auratus*	XP_026142923.1
Cc-CPT1	*Cyprinus carpio*	XP_042600342.1
Rt-CPT1	*Rana temporaria*	XP_040199083.1
Bb-CPT1	*Bufo bufo*	XP_040269509.1
Fr-CPT1	*Falco rusticolus*	XP_037246062.1
Cl-CPT1	*Columba livia*	XP_064905500.1
Pt-CPT1X1	*Pseudonaja textilis*	XP_026577711.1
Hb-CPT1X2	*Heteronotia binoei*	XP_060102043.1
Hs-CPT1	*Homo sapiens*	NP_689451.1
Bt-CPT1	*Bos taurus*	NP_001029521.1
Mm-CPT1	*Mus musculus*	NP_034078.2
Rn-CPT1X1	*Rattus norvegicus*	XP_006242242.1

Table S4: Species and their sequence numbers in Figure 7.

Abbreviated names	Full names	Protein ID
Sc-CPT1B	*Siniperca chuatsi*	
Sa-CPT1X1	*Scatophagus argus*	XP_046249180.1
Lc-CPT1X1	*Larimichthys crocea*	XP_019111118.2
Sa-CPT1	*Sparus aurata*	XP_030280465.1
Oa-CPT1	*Oreochromis aureus*	XP_031593118.1
Pf-CPT1	*Perca fluviatilis*	XP_039663911.1
Ms-CPT1	*Micropterus salmoides*	XP_038561324.1
Xg-CPT1X1	*Xiphias gladius*	XP_039988951.1
Sm-CPT1	*Scophthalmus maximus*	XP_035498774.1
Hh-CPT1	*Hippoglossus hippoglossus*	XP_034443988.1
Ma-CPT1	*Monopterus albus*	XP_020468078.1
Ci-CPT1	*Ctenopharyngodon idella*	XP_051726441.1
Ca-CPT1	*Carassius auratus*	XP_026142923.1
Ss-CPT1X1	*Salmo salar*	XP_014003915.2
Cc-CPT1	*Coregonus clupeaformis*	XP_041693535.1
Dr-CPT1	*Danio rerio*	NP_001315121.1
Cc-CPT1	*Cyprinus carpio*	XP_042600342.1
Rt-CPT1	*Rana temporaria*	XP_040199083.1
Bb-CPT1	*Bufo bufo*	XP_040269509.1
Fr-CPT1	*Falco rusticolus*	XP_037246062.1
Cl-CPT1	*Columba livia*	XP_064905500.1
Hs-CPT1	*Homo sapiens*	NP_689451.1
Bt-CPT1	*Bos taurus*	NP_001029521.1
Mm-CPT1	*Mus musculus*	NP_034078.2
Rn-CPT1X1	*Rattus norvegicus*	XP_006242242.1
Pt-CPT1X1	*Pseudonaja textilis*	XP_026577711.1
Hb-CPT1X2	*Heteronotia binoei*	XP_060102043.1
